# Peptide-Au Clusters Induced Tumor Cells Apoptosis via Targeting Glutathione Peroxidase-1: The Molecular Dynamics Assisted Experimental Studies

**DOI:** 10.1038/s41598-017-00278-6

**Published:** 2017-03-09

**Authors:** Meiqing Liu, Liang Gao, Lina Zhao, Jian He, Qing Yuan, Peng Zhang, Yawei Zhao, Xueyun Gao

**Affiliations:** 1 CAS Key Laboratory for Biomedical Effects of Nanomaterials and Nanosafety, Institute of High Energy Physics, Chinese Academy of Sciences, Beijing, 100049 China; 2 University of Chinese Academy of Sciences, Beijing, 100049 China; 3 Department of Chemistry and Chemical Engineering, Beijing University of Technology, Beijing, 100124 China

## Abstract

The original motivation of the article is to give a systematic investigation on the protocol of combining computer simulation and accurate synthesis of serial peptide protected gold clusters for potent tumor targeting therapy. Glutathione peroxidase-1 (GPx-1) is a crucial antioxidant selenoenzyme that regulates cellular redox level, thus becomes a potential target in cancer treatment. We firstly utilize molecular dynamic (MD) simulation to rationally design and screen serial peptide-Au cluster compounds with special peptide sequences and precise gold atoms, which can recognize and bind specific domain of GPx-1 with high affinity. The theoretical simulations were further verified by the following peptide-Au clusters synthesis and GPx-1 activity suppression studies in buffer and cells, respectively. Further cytological experiments corroborated that peptide-Au clusters are promising nanoparticles inducing tumor cells apoptosis by suppressing GPx-1 activity and increasing higher cellular reactive oxygen species level to initiate tumor cell apoptosis through intrinsic mitochondrial pathway.

## Introduction

After two decades of studies, nanoparticles are still be described by shape, size, composition and surface coating, etc. The versatile physicochemical and biological properties of nanoparticles are dependent on these basic parameters, therefore tailoring the parameters of nanoparticles would provide potential advanced materials in catalysis, energy and biomedicine field^1–3^. To date, however, nanoparticles could not be described as chemical molecules with precise molecular formula, uniform compositions and exact properties. Nowadays, it is widely accepted that precise synthesis of nanoparticles with well-defined molecular structure and consistent properties is the priority to ensure their intrinsic properties and relevant applications^4–6^. Although great success has been achieved in past two decades, most of synthetic strategies of nanoparticles refer to “random” and “try-and-error” processes. Meanwhile, nanoparticles are normally approximately characterized by aforementioned parameters and further applied in catalysis, energy, and biomedicine, leading to the versatile rather than uniform results in literatures^7, 8^. For instance in nanomedicine field, unlike natural macromolecules or organic molecules, nanoparticles are not capable of precisely targeting to the therapeutic aim in cells^9^. Because it is difficult to synthesize and describe these nanoparticles with precise molecular formula and consistent properties, thus these nanoparticles could not strictly recognize and bind the active site of the biomolecules following ‘lock and key’ molecular interaction mode^10, 11^. To explore nanoparticles as efficient inhibitors of therapeutic target, it is highly urgent to rationally design and elaborately synthesize nanoparticles with strict molecular structure, uniform chemical composition and consistent properties.

Metal clusters are molecular species composed of several to a few hundreds of metal atoms, which are intermediate states of matter between isolated molecules and large nanoparticles^12, 13^. Peptide-protected metal clusters possess a great deal of exceptional advantages such as accurate chemical formula, nanometer dimension, high photostability, good biocompatibility and scalable production^14, 15^. Nevertheless, it is a great challenge to achieve peptides with rational composition, sequence and length for precise synthesis of metal clusters with desirable structure, size, charge and targeting ability. Molecular dynamics (MD) simulations have been performed to study the precise conformations of molecules and interaction mechanism between specific molecules^16, 17^. It could be expected that the combination of computer simulation and accurate preparation would be a highly efficient approach to fabricate peptide-conjugated metal clusters for potent targeted therapy.

Glutathione peroxidase-1 (GPx-1), a crucial antioxidant selenoenzyme in mammalian cells, is selected as a model drug target for tumor treatment^18^. Suppression of intracellular GPx-1 activity induces the accumulation of hydrogen peroxide (reactive oxygen species, ROS), which would render tumor cells susceptible to ROS-induced apoptosis^19, 20^. Recent reports have witnessed gold-contained compounds such as auranofin and gold thiomalate can inhibit GPx-1 activity through interacting with selenocystein (Sec) by forming a stable gold-Sec complex to block the active site of the protein^21, 22^. In light of the well defined structure of mammalian GPx-1 consisting of four identical subunits^23^ and Sec is surrounded by some positively charged amino acid residues. With the aid of MD simulations, we attempt to design and synthesize a class of Au clusters with well-defined molecular structure consisting of exact number of peptides and Au atoms. It is anticipated that the peptides would recognize and bind the domain around the Sec site of GPx-1, thus Au cores would be more prone to interact with active Sec to suppress the enzyme activity with high efficiency.

Herein, we firstly designed a series of negative charged peptide-Au clusters to recognize and bind the domain around the Sec of GPx-1 by MD approach. After MD optimizations, these peptide-Au clusters can be well scored via comparing their recognition and bind affinity to GPx-1 throughout salt bridges, hydrogen bonds and hydrophobic interactions between the peptides and GPx-1. Next, these peptide-cluster compounds were chemically synthesized and their suppression activities were verified by studying GPx-1 activity in buffer solution and tumor cells, respectively. At last, the optimized peptide-Au clusters inducing lung tumor cells apoptosis via GPx-1 triggered intrinsic mitochondrial pathway was disclosed *in vitro*.

## Results

### Design of peptide-Au clusters

In the molecular structure design of peptide-Au clusters, we mainly considered the peptide sequences and the definite Au cluster size by checking the surficial electrostatic potential distribution and the concave structure around the Sec of GPx-1, respectively (Figure S1). For the peptide sequence design, we tried to choose highly negatively charged peptide with E (Glu) and D (Asp) to push Au cluster to approach the Sec of GPx-1 by strong electrostatic interaction^24^, this because the positive electrostatic potential mainly distributed around the active sites. We also introduced V (Val) and P (Pho) hydrophobic residues around C-terminal to bind with the uncharged surficial residues distributed about 15.0 Å away from the Sec of GPx-1. Meanwhile, CCY (Cys-Cys-Tyr) was added in peptide N-terminal to mineralize Au(III) and anchored Au cluster *in situ* ^25^. The P and G (Gly) were introduced into sequence to enhance the peptide flexibility to benefit peptide-Au cluster well matching and complex with GPx-1. Considering all these factors, we proposed three sequences including P1 = CCYGGPEEEEEVG (−5e), P2 = CCYGGPDDDEDVG (−5e) and P3 = CCYGGPEEVEEVG (−4e). In these candidates, the electrostatic and hydrophobic interaction are the main screen mechanisms, i.e. P1 and P2 are designed with different charges from P3, and the hydrophobicity of P1 and P2 are regulated by the different hydrophobic side chain lengths of E and D. For the Au cluster size selection, we built a serial of peptide protected Au clusters as Au_10_Peptide_5_, Au_25_Peptide_9_ and Au_40_Peptide_12_ with different sizes according to the typical thiolate coated Au clusters^26–28^. After measuring the dynamic diameters of Au clusters, we found the average diameters of Au clusters are 13.7 Å, 18.5 Å and 23.0 Å for Au_10_Peptide_5_, Au_25_Peptide_9_ and Au_40_Peptide_12_, respectively (see Figure S2). Thus, we figure out Au_25_Peptide_9_ as the molecular composition for the designed peptide protected Au cluster with diameter of 18.5 Å, which could match and dock the 20.7 Å concave width of Se active site well. Because the Au/S ratio is 25:18 in the coated Au_25_ cluster^29^, there should be nine peptides with 18 thiols coating one Au_25_ cluster in the above peptide-Au cluster design. The molecular structures can be built into Au_25_P1_9_, Au_25_P2_9_ and Au_25_P3_9_.

### Molecular dynamic simulation of molecular interactions

**Figure 1:**
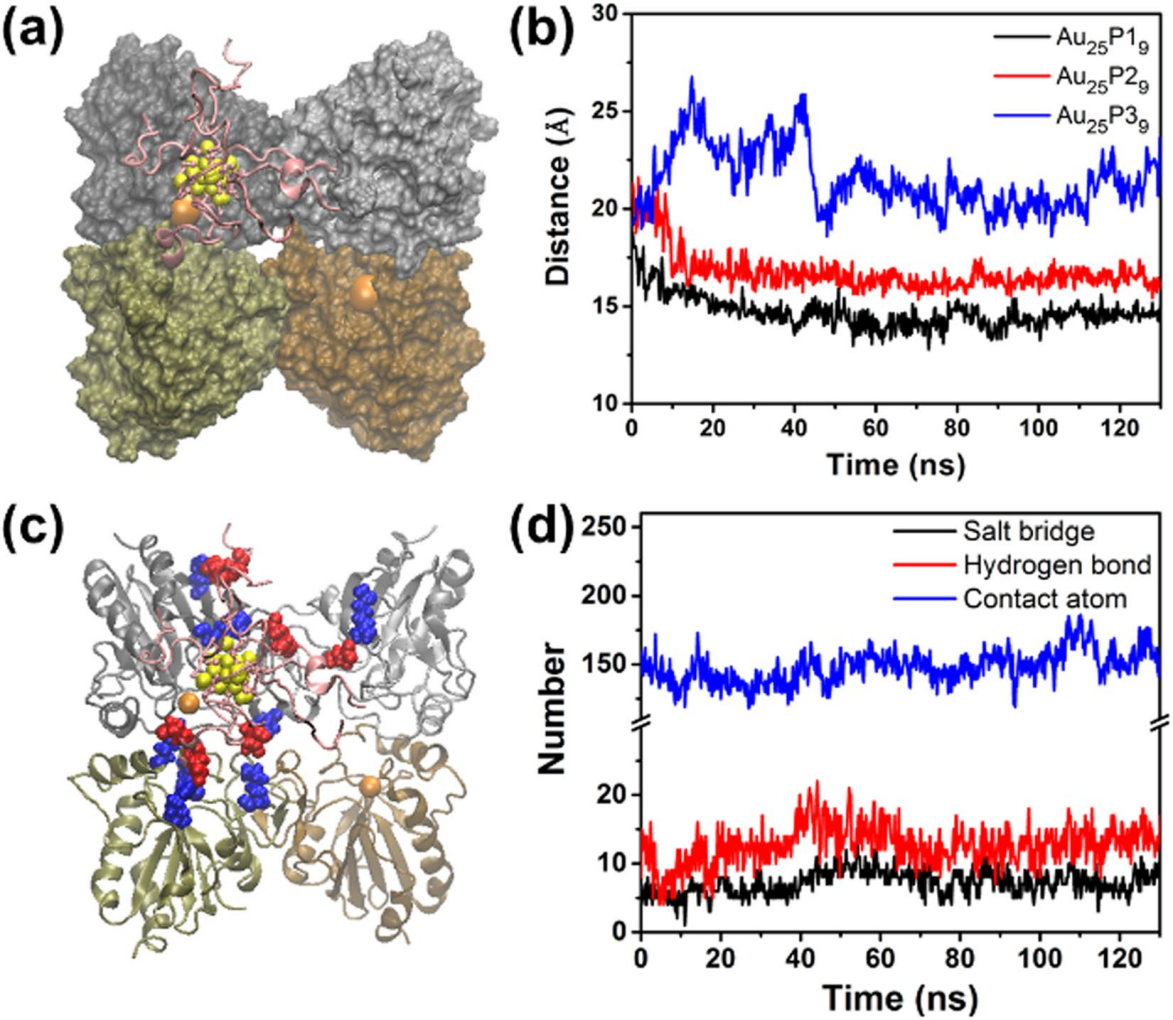
Comparison of GPx-1 binding abilities of Au_25_P1_9_, Au_25_P2_9_ and Au_25_P3_9_ candidates. (**a**) The stable configuration of designed Au_25_P1_9_ bound to the active site in subunit A of GPx-1 (at the 126^th^ ns in MD simulation). (**b**) Distance between the Se atom of Sec45 (active site in subunit A) and the Au_25_ core COM of Au_25_P1_9_ (black), Au_25_P2_9_ (red) and Au_25_P3_9_ (blue) during their binding dynamics to GPx-1. (**c**) Salt bridge distribution between designed Au_25_P1_9_ and GPx-1 surface in the stable configuration (at the 126^th^ ns in MD simulation). Positive and negative charged residues are in blue and red, respectively. (**d**) The number of salt bridges (black), hydrogen bonds (red) and contact atoms (blue) between designed Au_25_P1_9_ and GPx-1 surface.

Subsequently, to screen the most effective peptide-Au cluster structrure, we investigated the dynamic behaviors of the three peptide-Au cluster candidates during their molecular interactions with the active site of GPx-1. Utilizing the molecular docking method, we investigated the possible interaction sites of Au_25_P1_9_, Au_25_P2_9_ and Au_25_P3_9_ in their representative configurations (Figure S3) and found that they all can approach to the Sec active site of GPx-1. We took the Sec active site in subunit A as the targeting site and set the same initial center of mass (COM) distance as 20.0 Å between Se atom of Sec45 (center of active site) and Au_25_ core in peptide-Au cluster candidates, respectively. The subunits in upper left, lower left, upper right and lower right of GPx-1 were denoted as A, B, C and D. Then, we performed MD simulations of the interaction dynamic procedures between peptide coated Au_25_ candidates and the active site to screen their targeting abilities. After the above screening procedures, we filtered Au_25_P1_9_ as the valid targeted compound with a stable binding configuration to block the Sec active site of GPx-1 (Fig. 1a). The targeted binding abilities of candidates can be clearly compared by their COM distance to Se atom in Sec active site center during the interaction dynamics (Fig. 1b). The candidates start from the same location, but evolve into different ways during the simulations. In detail, Au_25_P1_9_ (black) quickly approaches to the center of active site and stably binds to the active site with 14.5 Å COM distance. Au_25_P2_9_ (red) fluctuates around the starting location in the first 10 ns, then moves near to the active site at about 16.5 Å COM distance. As for Au_25_P3_9_ (blue), it diffuses away from the active site region during the first 50 ns, and locates on the protein surface around 22.0 Å COM distance to the active site. Therefore, it is clear to figure out the best targeted agent is Au_25_P1_9_. To better understand the effective targeting ability of Au_25_P1_9_, we took a close look at its salt bridge interaction mode in the stable binding configuration at the 126^th^ ns (Fig. 1c). There are 10 salt bridges formed between four coating peptides (denoted as 1 to 4) of Au_25_P1_9_ and GPx-1 surface, i.e. Glu408-Arg173, Glu406-Arg173 between peptide 1 and GPx-1 subunit A; Glu410-Arg18, Glu408-Lys84, C-terminal Gly412-Lys84, C-terminal Gly412-Lys110 between peptide 2 and GPx-1 subunit B; Glu406-Lys144 between peptide 3 and Gpx-1 subunit A; Glu406-Lys117 between peptide 3 and GPx-1 subunit B; Glu407-Arg178 between peptide 4 and GPx-1 subunit A; Glu410-Arg10 between peptide 4 and GPx-1 subunit C. In Fig. 1d, the average numbers of salt bridges and hydrogen bonds are 8 and 13 between Au_25_P1_9_ and GPx-1 surface during the stable binding (50–130 ns), respectively. The correlation contact atoms averagely reach 153. As a result, the designed Au_25_P1_9_ can stably bind to the active site of GPx-1 due to the sufficient and stable salt bridge and hydrogen bond interactions. The detail binding simulations of Au_25_P2_9_ and Au_25_P3_9_ candidates were analyzed in Figure S4. As the screening mechanism proposed above, we can filter Au_25_P1_9_ and Au_25_P2_9_ with approaching to charged active site by electrostatic interaction and further figure out Au_25_P1_9_ with longer hydrophobic side chain of E interact with uncharged residues distributed around the Sec active site (see Figure S5).

### Synthesis and characterization of peptide-Au clusters

**Figure 2:**
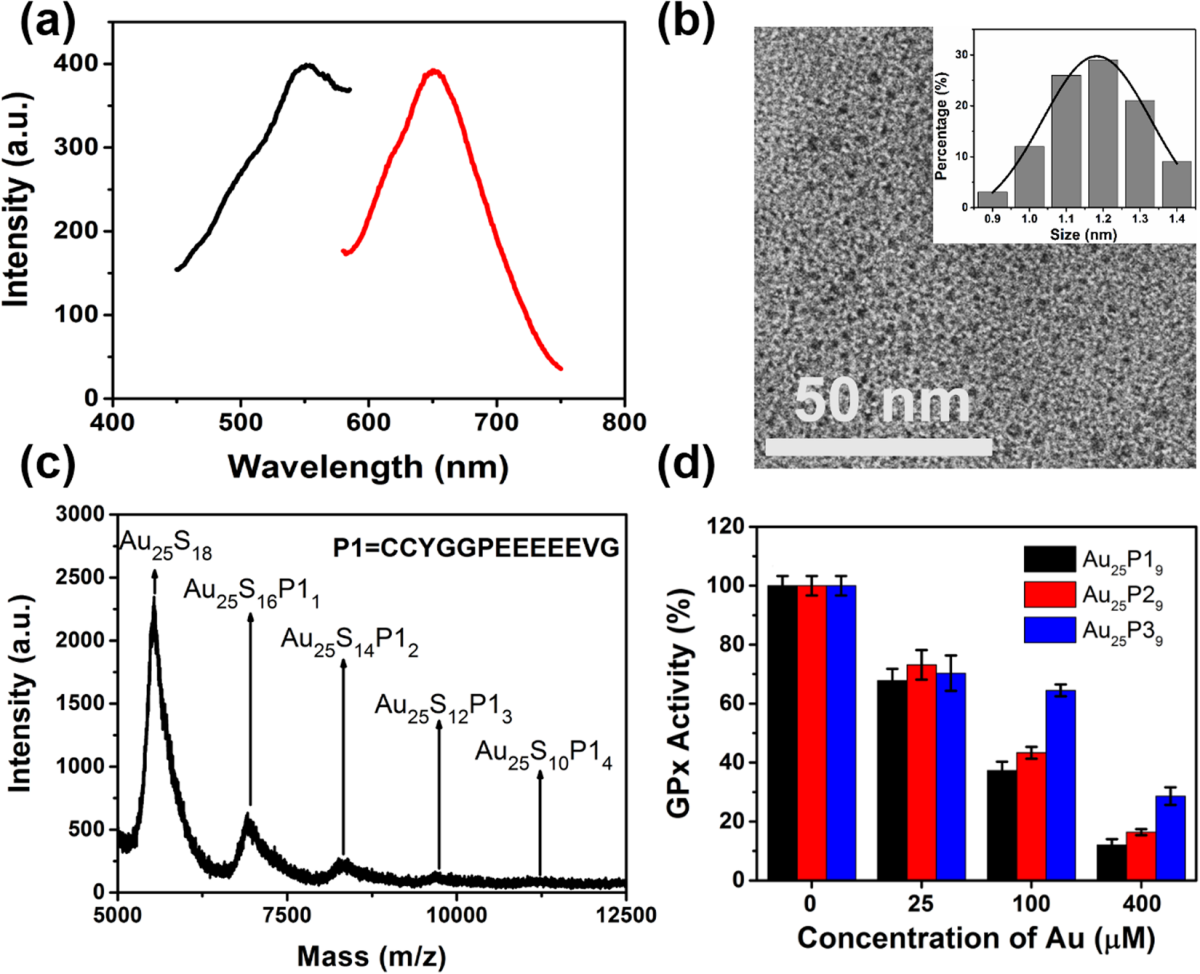
(**a**) Fluorescence excitation (black line) and emission (red line) spectra of Au_25_P1_9_. (**b**) HRTEM images of Au_25_P1_9_. Inset shows diameter distribution from statistic results of 100 particles. (**c**) MALDI-TOF MS spectra of Au_25_P1_9_. (**d**) GPx-1 activity suppressed by peptide-Au clusters in a dose dependent manner in buffer.

To verify the results of molecular simulations, these peptide-Au clusters were precisely synthesized by chemical method. The optical properties of these peptide-Au clusters are similar to previously reported Au_25_ clusters^25, 30–34^. As shown in Figure S7, peptide-Au clusters depict obvious peptide absorption peaks at around 275 nm and are with a gradient absorption from 350 to 600 nm ascribed to Au cluster. All the fluorescence spectra of peptide-Au clusters present a maximum excitation peak at 550 nm and a maximum emission peak at 650 nm, whose red emission can be used to track their location in tumor cells in an expedient manner (Fig. 2a, Figures S6 and S8). HRTEM images show that they are well dispersed with diameter of ~1.15 nm (Fig. 2b and Figure S9), whose size are close to Fermi wavelength thus cause these unique optical properties^35^. To determine the accurate molecular formula of these peptide-Au clusters, matrix-assisted laser desorption/ionization time of flight mass spectrometry (MALDI-TOF MS) was further employed. In Fig. 2c and Figure S10, the mass spectra of these peptide-Au clusters are composed of a series of peaks (black arrows) between 5.0–12.5 k m/z and the spacing between adjacent peaks is equal to one peptide with two thiol groups missing, this is attributed to the C–S bond can be easily broken during the desorption/ionization laser processing^36^. Since the strongest peak of these peptide-Au clusters locate at about 5501 m/z and the m/z of the main peaks match the formula Au_25_S_18-2m_P_m_ (where P is peptide, m = 0–4), the molecular formula of synthesized peptide-Au clusters can be assigned to Au_25_P_9_. The identification of molecular formula indicates that these peptide-Au clusters we designed and optimized by MD simulations have been successfully synthesized. In the following quantitative analysis, the concentration of these peptide-Au clusters can be measured by inductively coupled plasma mass spectrometry (ICP-MS) (see calibration curve of Au standards in Figure S11).

### GPx-1 enzyme activity suppression by peptide-Au clusters in buffer

The peptide-Au clusters suppress GPx-1 enzyme activity were studied in buffer solution. The experimental results reveal that pure GPx-1 activity is suppressed by peptide-Au clusters in dose dependent manner (Fig. 2d and Figure S12). At an Au dose of 400 μM, GPx-1 activity is 12%, 16% and 29% when treated by Au_25_P1_9_, Au_25_P2_9_ and Au_25_P3_9_, respectively. The experiments match the MD simulations exactly where the Au_25_P1_9_ exhibits the best suppression capacity for GPx-1.

### GPx-1 enzyme activity suppression and ROS generation in cells

**Figure 3:**
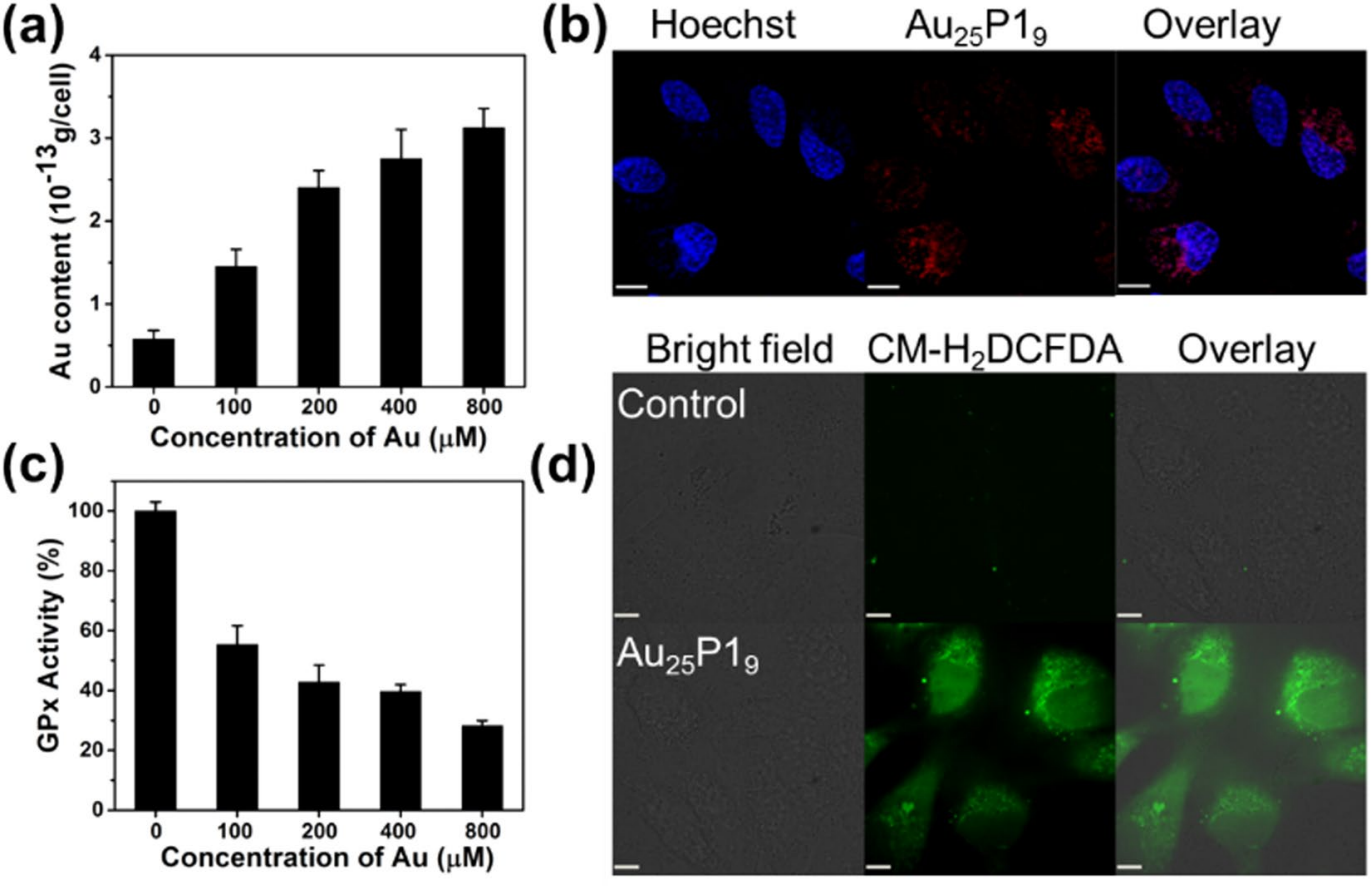
(**a**) Quantification of Au_25_P1_9_ uptake by A549 cells (**b**) CLSM images of A549 cells treated with 800 µM of Au_25_P1_9_ for 48 h and the cell nuclei were stained with Hoechst 33342. (**c**) GPx-1 activity suppressed by Au_25_P1_9_ in a dose dependent manner in A549 cells. (**d**) CLSM images of cellular ROS level of A549 cells treated with Au_25_P1_9_ for 48 h, Scale bar is ~11 µm.

As the peptide-Au clusters could well suppressing the GPx-1 activity in buffer solution, we further check if the peptide-Au clusters could be taken into human non-small cell lung carcinoma cells (A549 cells), suppress the GPx-1 and further up-regulate the ROS level in cell. As shown in Fig. 3a,b and Figure S13, cellular uptake of Au_25_P1_9_ follow a dosage dependent manner and their red emission can be observed in the cytoplasm of live A549 cells by confocal laser scanning microscopy (CLSM). These results imply that Au_25_P1_9_ located in cell may interact with GPx-1 therein. To verify this assumption, we firstly detected the GPx-1 expression level in A549 cells after treated with Au_25_P1_9_ for 48 h. The result of western blotting analysis show that GPx-1 level remains stable when cells treated with different doses of Au_25_P1_9_ (Figure S17). Meanwhile, the cellular GPx-1 activities of these samples were measured. The cellular GPx-1 activity significantly decreases as Au_25_P1_9_ dose increase in cell cytosol (Fig. 3c). In particular, cellular GPx-1 activity decreases to 27% after cells are treated with 800 μM of Au. For the control experiments, Au_25_P1_9_ was added into the cell lysate containing GPx-1. As shown in Figure S14, the GPx-1 activity is also suppressed via an Au dose dependent manner.

Considering the suppression of cytoplasm GPx-1 activity could enhance the accumulation of ROS, we investigated intracellular ROS level in Au_25_P1_9_ treated A549 cells by CLSM and flow cytometry. As shown in CLSM images (Fig. 3d), compared to control, Au_25_P1_9_ treated cells stained by CM-H2DCFDA exhibit obvious green fluorescence, a sign of increased intracellular ROS. Quantitatively, when cells were treated by Au_25_P1_9_ with Au dose of 800 μM, the intracellular ROS level increases to ~210% by flow cytometry analysis (Figure S15). Such higher ROS level is expected to trigger cell apoptosis.

### Cell apoptosis

**Figure 4:**
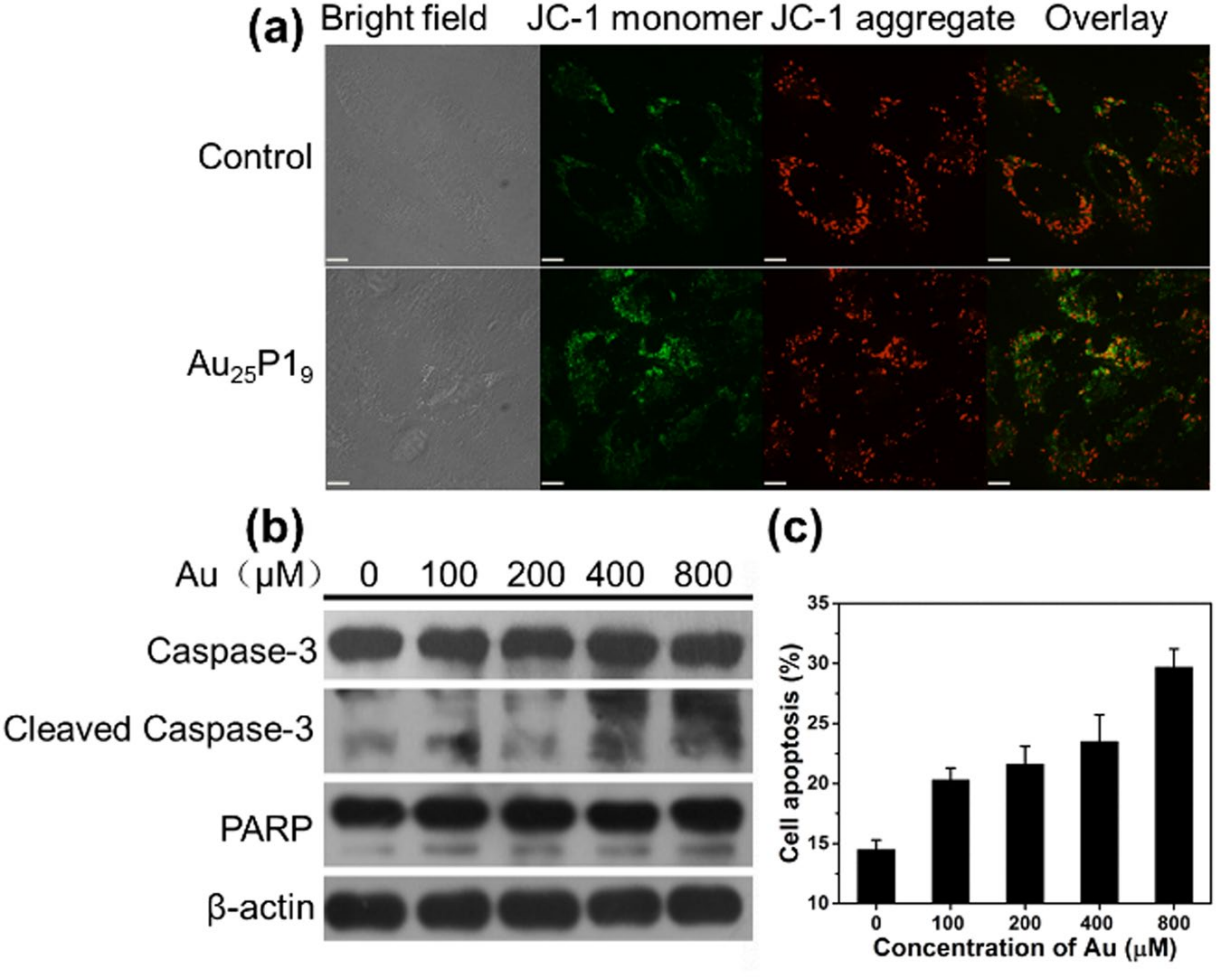
(**a**) CLSM images of mitochondrial membrane potential (JC-1 staining) in A549 cells pre-treated with Au_25_P1_9_ at an Au dose of 800 μM for 48 h, Scale bar is ~11 μm. (**b**) The express level of caspase-3, PARP and β-actin extracted from A549 cells pre-treated with serial doses of Au_25_P1_9_ for 48 h. (**c**) A549 cells apoptosis induced by Au_25_P1_9_ in a dose-dependent manner.

Generally, the significant increase of intracellular ROS level could further induce cell apoptosis via intrinsic mitochondrial pathway^37, 38^. In mitochondrial pathway, ROS would decline mitochondrial membrane potential and initiate a cascade process that series of biomolecules will be released from mitochondria into the cytosol to activate downstream biomolecules including caspase-3, followed by the activated caspase-3 cleaving poly(ADP-ribose) polymerase (PARP) to induce cell apoptosis^39^. To prove this, the change of mitochondrial membrane potential in Au_25_P1_9_ treated A549 cells was firstly analyzed by live cell fluorescent dye 5, 5′, 6′, 6′-tetrachloro-1, 1′, 3, 3′-tetraethylbenzimidazolocarbocyanine iodide (JC-1). As shown in Fig. 4a, a red to green emission shift can be observed in most cells after treated with Au_25_P1_9_, indicating mitochondria depolarization. For further studying, A549 cells were treated with Au_25_P1_9_ for 48 h at different doses, respectively. Then the cells were lysed and caspase-3 and PARP were extracted for immunoblotting studies. The results in Fig. 4b disclose that Au_25_P1_9_ lead to an Au dose-dependent increase of cleaved caspases-3 and cleaved PARP levels in A549 cells. In addition, the activation of caspase-3 was also monitored and verified by the strong Nucview488 emission in cell imaging by CLSM (Figure S16). Above mentioned results indicate cell apoptosis via mitochondrial pathway happens when tumor cells are treated with Au_25_P1_9_. Further, we investigated the cell apoptosis ratio by Annexin V-FITC/PI dual staining assay. The data show that the cell apoptosis continuously increases from 5.8% to 15.2% when the Au_25_P1_9_ dose increases from 100 μM to 800 μM (Fig. 4c and Figure S18). In addition, Au_25_P1_9_, rather than free peptides, can suppress cell viability which further supports peptide-Au clusters induced cell apoptosis (Figure S19a and S19b). We also considered the cell toxicity differences of Au_25_P1_9_ in other two tumor cell types as a control. Hela and HCT-8 cell lines were treated with Au_25_P1_9_ under the same condition as that of A549 cells line. The change of the cell viability of two cell lines was almost negligible (Figure S19c). This result indicates that the gold clusters were more effective to induce A549 cell apoptosis, which may result from the different susceptibility to oxidative stress^40, 41^. As hypothesis, Au_25_P1_9_ induce A549 cells apoptosis via intrinsic mitochondrial pathway, because Au_25_P1_9_ suppress GPx-1 activity and induce ROS level significant enhancement in cytosol.

## Discussion

Computer-Aided Drug Design is a greatly important method to develop candidate drug for potent targeting therapy. In the molecular structure design of peptide-Au cluster, we mainly considered the peptide sequences and definite Au cluster size by checking the surficial electrostatic potential distribution and the concave structure around the Sec of GPx-1. Through MD simulations of molecular interactions between three peptide-Au cluster candidates and the active site of GPx-1, we filtered Au_25_P1_9_ as the valid targeted compound with stable binding to the active site of GPx-1, which could be mainly attributed to their efficient electrostatic and hydrophobic interaction. In the following experimental studies, we successfully synthesized peptide-Au clusters and found Au_25_P1_9_ was the most effective candidate for suppression of GPx-1 activity in buffer and cells.

In summary, under the guidance of MD stimulations, we rationally design and facilely synthesize peptide-Au clusters with strict molecular structure and consistent properties as potential anticancer drugs targeting GPx-1. As optimization by MD simulations and following experimental studies, the peptide shell in the peptide-Au clusters can recognize and bind GPx-1 with high affinity, thus the Au core with matched size could be more prone to interact with active Sec to efficiently suppress its activity. As a result, peptide-Au clusters as conceived and prepared can mediate tumor cells apoptosis via ROS induced mitochondrial pathway. The strategy paves a new route to develop other anticancer drugs targeting specific proteins following “lock and key” molecular interaction mode.

## Methods

### Materials

The peptides (N_2_H-CCYGGPEEEEEVG-COOH (named P1), NH_2_-CCYGGPDDDEDVG-COOH (named P2) and NH_2_-CCYGGPEEVEEVG-COOH (named P3)) were chemically synthesized by a solid phase method (China Peptides Co. Ltd, Purity: 98%). Hydrogen tetrachloroaurate (III) (HAuCl_4_·4H_2_O) was purchased from Sinopharm Chemical Reagent Co. Ltd. Sodium hydroxide (NaOH), nitric acid (HNO_3_), hydrochloric acid (HCl) and hydrogen peroxide (H_2_O_2_) were purchased from Beijing Chemical Reagent Co., China. Human non-small cell lung carcinoma cells (A549 cells), Henrietta Lacks strain of cancer cells (Hela cells) and human colon adenocarcinoma cells (HCT-8 cells) were from Cancer Institute and Hospital, Chinese Academy of Medical Sciences. Fetal bovine serum (FBS) and trypsin-EDTA were obtained from Gibco, USA. Penicillin-streptomycin solution, cell culture medium DMEM and RPMI 1640 were purchased from Hyclone. Hoechst 33342, LysoTracker®Green DND-26, and CM-H_2_DCFDA were from Molecular Probes, USA. NucView™ 488 Caspase-3 Assay Kit was bought from Biotium, USA. 5,5′,6,6′-tetrachloro-1,1′,3,3′-tetraethylbenzimidazolocarbocyanine iodide (JC-1), cell lysis buffer for Western and IP, phenylmethanesulfonyl fluoride (PMSF) and BCA Protein Assay Kit were obtained from Beyotime Institute of Biotechnology, China. Protease inhibitor cocktail tablets were from Roche Diagnostics, GmbH, Mannheim, Germany. Cell counting kit-8 (CCK-8) reagents and Annexin V-FITC/Propidium (PI) Apoptosis Detection Kit were purchased from Dojindo Laboratories, Japan. GPx from bovine erythrocytes from rat liver was provided by Sigma Aldrich, USA. GPx Activity Colorimetric Assay Kit was provided by Biovision, USA. Anti-GPx-1 antibody was offered by Abcam, UK. PARP antibody, caspase-3 antibody and β-actin antibody were offered by Cell Signaling Technology, USA. Amicon ultra centrifugal filter devices (Merck Millipore, MWCO: 10 kDa) were used for purification steps. Ultrapure Millipore (Mini-Q) water (18.2 MΩ) was used throughout the experiments. All glassware was thoroughly washed with aqua regia (conc. HNO_3_: conc. HCl, volume ratio = 1:3), rinsed with ultrapure water and ethanol, and then dried in an oven prior to use. All other materials were commercially available and used as received unless otherwise mentioned.

### Molecular simulation methods

(1) Molecular dynamics (MD) simulation. The structure of GPx-1 was obtained from the Protein Data Bank (PDB ID: 1GP1)^42^. The relaxation system for GPx-1 tetramer contained ~120 000 atoms in 105 Å × 105 Å × 112 Å periodic simulation box. Each of the peptide-Au cluster candidates was respectively relaxed in the dimension as 81 Å × 81 Å × 90 Å containing ~60 000 atoms. We studied the binding dynamics of Au_25_P1_9_, Au_25_P2_9_ and Au_25_P3_9_ candidates to GPx-1 in 121 Å × 121 Å × 130 Å dimension containing ~180 000 atoms, respectively. The TIP3P water was used in all systems at a neutral physiological ionic concentration as 0.15 M. After energy minimization, the binding interactions between Au_25_P1_9_, Au_25_P2_9_, Au_25_P3_9_ candidates and GPx-1 were simulated with the 130 ns production run for each binding system. These MD calculations were performed in NPT ensemble at 1 bar and 310 K. The pressure and temperature were maintained by the Parrinello−Rahman barostat and the velocity rescaling thermostat. The AMBER99SB force field in CHARMM format was employed in this study. The parameter of Se was referenced from the previous study^43^. The particle-mesh Ewald (PME) method was used to calculate long-range electrostatic interactions. A typical 12 Å cutoff distance was applied to calculate van der Waals and real-space Coulomb interactions. A time step of 2 fs was utilized. All the simulations were carried out using NAMD 2.8^44^. (2) Molecular docking simulation. We used ZDOCK package^45^ to study the possible interaction sites of Au_25_P1_9_, Au_25_P2_9_ and Au_25_P3_9_ in their representative configurations (Figure S3) binding to GPx-1 surface. All the peptide-Au cluster candidates can approach to the Sec active site of GPx-1. The ZDOCK sever is at the website as http://zdock.umassmed.edu.

### Synthesis of peptide-Au clusters

Peptide-Au clusters were synthesized by a previous method with a slight modification^25^. The peptide (2.5 mg) was dissolved in NaOH solution (16.61 mM, 1.505 mL). Subsequently, an aqueous solution of HAuCl_4_ (25 mM, 75 μL) was slowly added under vigorous stirring and then NaOH (0.5 M, 125 μL) was introduced for another 5 min. Finally, the mixture was stored in dark for 24 h to produce the peptide-Au clusters products. The as-synthesized products were purified through ultrafiltration tube (Millipore, MWCO: 10 KDa) to remove free ions and peptide. The purified products were stored in dark at 4 °C for further experiments.

### Characterization of peptide-Au clusters

UV-Vis absorption spectra of peptide-Au clusters were recorded by a spectrophotometer (Shimadzu UV-1800, Japan). Fluorescence spectra of peptide-Au clusters were obtained by a fluorescence spectrophotometer (Shimadzu RF-5301, Japan). The size distribution of synthesized peptide-Au clusters was characterized by HRTEM. Samples were prepared by casting and evaporation a droplet of water solution on a 300-mesh holey carbon-coated copper grid (Electron Microscopy Sciences, Washington, USA). Then the high resolution images were acquired using a HRTEM (TECNAI F20, USA) with an operating voltage of 200 kV. The molecular formula of peptide-Au clusters was analyzed by MOLDI-TOF MS (UltrafleXtreme, Germany) on an ABI MALDI-TOF system in positive ion linear mode using α-cyano-4-hydroxycinnamic acid (CHCA) as the matrix.

### Concentration of peptide-Au clusters analyzed by ICP-MS

Au concentration was measured by ICP-MS (Thermo Elemental X7, USA). The prepared and purified 50 µL peptide-Au clusters solutions was predigested by HNO_3_ and H_2_O_2_ at a volume ratio of 3:1 for 12 h and further digested by aqua regia at 160 °C. When evaporated to 0.5 mL, the sample was diluted to 5000-fold by 1% HCl and 2% HNO_3._ Calibration plots for standard aqueous Au were obtained by injecting a series of standard aqueous Au solutions (0.5, 1, 5, 10, 50 μg/L containing 1% HCl and 2% HNO_3_) into the ICP-MS system. A 20 ng/mL bismuth standard solution containing 1% HCl and 2% HNO_3_ was injected as an internal standard. The completely digested and diluted sample was injected to measure the Au content. The experiment was carried out in triplicate. The Au concentration could be accurately quantified by calibration curve.

### Activity assay of GPx-1 in buffer solution

Pure GPx (lyophilized powder, ≥300 units/mg protein) were diluted by GPx sample buffer and incubated with peptide-Au clusters (Au_25_P1_9_, Au_25_P2_9_ and Au_25_P3_9_) at a series of Au dose (0, 25, 100, 400 μM) for 10 min, respectively. Then the GPx-1 activity was assayed spectrophotometrically by GPx Activity Colorimetric Assay Kit following the manufacturer’s instructions. The GPx activity of untreated samples was set as 100%, and that of peptide-Au clusters treated samples were expressed as a relative percentage of untreated ones.

### Au_25_P1_9_ uptake determined by ICP-MS

The cells were cultured in medium supplemented with 10% fetal bovine serum and 1% penicillin and streptomycin in a culture flask (25 cm^2^), reseeded every 2–4 days to maintain subconfluency and kept in humidified atmosphere of 95% air and 5% CO_2_ at 37 °C. After cells reaching 80% confluence during the exponential growth in culture flask, the cells were harvested and subcultured on 6-well plate (at the density of 1 × 10^5^ cells/well) for 24 h. Then the medium was discarded and fresh culture medium with Au_25_P1_9_ at different doses was added to make cells exposed to final Au doses at 0, 100, 200, 400 and 800 μM, respectively. After another 48 h incubation, the cells were treated with trypsin and collected, and then washed thrice with PBS. 5 × 10^5^ cells of each sample counted by flow cytometry were predigested by HNO_3_ and H_2_O_2_ at a volume ratio of 3:1 for 12 h and further digested by aqua regia at 160 °C, and then diluted to 10 mL aqueous solution with 1% HCl and 2% HNO_3_. Finally, the amount of Au_25_P1_9_ taken into cells was measured by ICP-MS with Au calibration curve.

### Cellular location of Au_25_P1_9_ observed by CLSM

A549 cells were seeded on glass-bottom dishes (35 mm, MatTek Corporation) for confocal laser scanning microscopy (CLSM) observation. After 24 h, Au_25_P1_9_ was added to culture medium and the final Au dose was 800 µM. The A549 cells were next exposed to Au_25_P1_9_ for 48 h. Then the cells were washed three times with PBS and incubated with 10 µg/mL Hoechst 33342 at 37 °C under darkness for 10 min. Before observation, the culture medium was discarded and the cells were washed three times with PBS, supplemented with fresh culture medium. Finally, in A549 cells, the cell nucleus and Au_25_P1_9_ were imaged under Nikon Ti-e microscope with excitation at wavelengths of 405 nm and 560 nm, respectively. To identify the accurate location of Au_25_P1_9_ in cytoplasm of A549 cells, lysotracker green, a lysosome probe, was added in the medium after cells were incubated with Au_25_P1_9_ for 48 h. The cells were then observed by CLSM with excitation at wavelengths of 488 nm and 560 nm, respectively.

### Activity assay of GPx-1 in A549 cells and cells lysate

A549 cells were prior seeded on 6-well plate (at the density of 1 × 10^5^ cells/well) for 24 h. Then the medium was discarded and fresh culture medium with Au_25_P1_9_ at different doses was added to make cells exposed to final Au doses at 0, 100, 200, 400 and 800 μM, respectively. After another 48 h incubation, the cells were washed thrice with PBS and lysed by cell lysis buffer containing 1 mM PMSF for 15 min on a shaker at 4 °C. The lysed cells were collected and centrifuged for 20 min at 12,000 g to get total protein of cells. The protein dose of each sample was quantified by BCA Protein Assay Kit and the GPx-1 activity of peptide-Au clusters treated A549 cells was measured by GPx Activity Colorimetric Assay Kit, respectively. For the control experiments, a series of Au doses of Au_25_P1_9_ at 0, 5, 25, 100, 400 μM were incubated with the GPx-1 containing cell lysate of A549 cells for 10 min and the GPx-1 activity of theses samples was also measured. The GPx-1 activity of untreated samples was set as 100%, and that of peptide-Au clusters treated samples were expressed as a relative percentage of untreated ones.

### Intracellular ROS level analyzed by CLSM and flow cytometry

For CLSM observation, cells were prior cultured on glass-bottom dishes. Au_25_P1_9_ was added to culture medium and the final Au dose was 800 µM. After 48 h. the cells were washed three times with PBS and incubated with 5 µM CM-H_2_DCFDA, at 37 °C under darkness for 30 min. Before CLSM observation, the culture medium was discarded and the cells were washed three times with PBS, supplemented with fresh culture medium. Finally, ROS in cells were highlighted by green fluorescence emission of CM-H_2_DCFDA, imaged under Nikon Ti-e microscope with excitation at wavelengths of 488 nm. For flow cytometry analysis, cells were prior seeded on 6-well plate (at the density of 1 × 10^5^ cells/well) for 24 h. Then the medium was discarded and fresh culture medium containing Au_25_P1_9_ with Au dose at 800 μM was added. For another 48 h incubation, the cells were washed thrice with PBS, and incubated with 5 µM CM-H_2_DCFDA at 37 °C under darkness for 30 min. After washing with PBS, the cells were collected and suspended in ice cold PBS. Fluorescence intensity of 20,000 events was recorded using an Accuri C6 flow cytometer and the level of intracellular ROS was analyzed by Cflow software.

### Mitochondrial membrane potential (ΔΨ_m_) assay

Disruption of ΔΨ_m_ is an important step in the induction of cellular apoptosis. JC-1, a sensitive mitochondrial dye, was used to evaluate the ΔΨ_m_. JC-1 can aggregates in the mitochondrial matrix to yield red fluorescence (λ_em_ = 590 nm) at high mitochondrial membrane potential, while JC-1 monomer has green fluorescence at low mitochondrial membrane potential (λ_em_ = 527 nm). Therefore, using JC-1 easily signals the loss of ΔΨ_m_, an event of early cell apoptosis. After treated with 800 µM of Au_25_P1_9_ for 48 h on glass-bottom dish, cells were stained with JC-1 at 37 °C for 20 min under darkness. Then, cells were washed twice with cold buffer solution and JC-1 fluorescence inside cells was immediately observed by CLSM. JC-1 monomers and JC-1 aggregates were observed with excitation at wavelengths of 488 nm.

### Caspase-3 activity assay

A549 cells were seeded on glass-bottom dishes for 24 h, and the Au_25_P1_9_ was added to culture medium and final Au dose was 800 µM. A549 cells were next exposed to Au_25_P1_9_ for 48 h. Then the cells were washed three times with PBS and incubated with 5 µM Nucview488 caspase-3 substrate (which can be cleaved by caspase-3 and release the DNA dye to stain DNA with bright green fluorescence during apoptosis) at room temperature for 30 min. Finally, cells were washed with PBS and observed by CLSM with excitation at wavelengths of 488 nm.

### Expression of GPx-1, caspase-3 and PARP analyzed by western blotting

The expression of GPx-1, caspase-3 and PARP from Au_25_P1_9_ treated A549 cells were analyzed by western blotting. A549 cells were prior seeded on 6-well plate (at the density of 1 × 10^5^ cells/well) for 24 h. Then the medium was discarded and fresh culture medium with Au_25_P1_9_ at different doses was added to make cells exposed to final Au doses at 0, 100, 200, 400 and 800 μM, respectively. After another 48 h incubation, the cells were harvested and lysed by cell lysis buffer for Western and IP (containing 20 mM Tris-HCl, pH7.5, 150 mM NaCl, 1% Triton X-100, sodium pyrophosphate, β-glycerophosphate, EDTA, Na_3_VO_4_ and leupeptin), protease inhibitor cocktail tablet and 1 mM PMSF for 15 min on a shaker at 4 °C. The lysed cells were collected and centrifuged for 15 min at 12,000 g to get total protein of cells. The protein dose of each sample was prior quantified, and then loading buffer was added. After boiling for 5 min, the protein samples with equal amount were separated on 12% SDS polyacrylamide gel electrophoresis and then transferred onto the polyvinylidene difluoride (PVDF) membrane. Membrane was blocked by 5% nonfat dried milk prepared in tris(hydroxymethyl)amin omethane-NaCl-Tween 20 (TBST) at room temperature for 1 h, and then incubated with primary antibodies specific to β-actin, Caspase-3, PARP and GPx-1 at 4 °C overnight, respectively. After that, horseradish peroxidase-conjugated secondary antibody was added at room temperature for 1 h, and then the protein bands from antibody-antigen reactions were visualized using Amersham ECL TM Prime Western Blotting Detection Reagent (GE healthcare, UK).

### Apoptosis evaluation

Apoptosis detection with Annexin V-FITC/PI fluorescence dual staining was carried out by flow cytometry. Briefly, about 2 mL of 1 × 10^5^ cells/mL of A549 cells were seeded in 6-well plate for 24 h before treatment with Au_25_P1_9_ at different Au doses. The cells were treated with Au_25_P1_9_ (Au dose at 0, 100, 200, 400 and 800 μM) for 48 h, respectively. Then culture medium and cells in each well were collected and centrifuged at 1200 rpm for 3 min. The supernatant was discarded and the cells were washed twice with PBS. 195 μL Annexin V-FITC binding buffer was added to re-suspend the cells at a dose of ~5 × 10^5^ cells/mL in 1.5 mL Eppendorf tube. 5 μL Annexin V-FITC and 10 μL PI stains were added into each cell suspension, respectively. The tubes were incubated at room temperature under darkness for exactly 15 min. The fluorescence of the cells was determined immediately with flow cytometer. Cells at the early stage of apoptotic process were stained with the Annexin V-FITC alone. Necrotic cells were stained by both the Annexin V-FITC and PI. Live cells showed no staining by either Annexin V-FITC or PI. So, the percentage of apoptotic and necrosis cells can be analyzed by Cflow software.

### Cell viability assay

Briefly, cells were prior cultured on 96-well plates (at the density of 5 × 10^3^ cells/well) for 24 h to allow cells attachment. The cells were treated with different doses of these free peptides (0, 1, 4, 16, 64, 256, 1024 µM) and peptide-Au clusters (0, 50, 100, 200, 400, 800 µM in Au dose) for 48 h, respectively. The cells were washed three times with PBS and then incubated with fresh growth medium containing 10% (v/v) CCK-8 reagent for 1 h at 37 °C for cell viability assay. The absorbance was measured at 450 nm by using a microplate reader (SpectraMAX M2, Sunnyvale, California). Cell viability of untreated cells was set as 100%, and that of treated cells were expressed as a relative percentage of untreated cells. All data were shown as mean percentages ± standard deviation from three independent experiments.

## Electronic supplementary material

Supporting information
