# Supplementary material for: Peptide-Au Clusters Induced Tumor Cells Apoptosis via Targeting Glutathione Peroxidase-1: The Molecular Dynamics Assisted Experimental Studies
Source: Sci Rep. 2017 Mar 9;7:131. doi: 10.1038/s41598-017-00278-6 (PMC5428013; doi:10.1038/s41598-017-00278-6)
Supplement: Supplementary file 1 — Supporting information [file 41598_2017_278_MOESM1_ESM.doc]

**Supporting information**

**Peptide-Au Clusters Induced Tumor Cells Apoptosis via Targeting Glutathione Peroxidase-1: The Molecular Dynamics Assisted Experimental Studies**

**Meiqing Liu^1,2^, Liang Gao^1,*^, Lina Zhao^1,*^, Jian He^1^, Qing Yuan^1^, Peng Zhang^1^, Yawei Zhao^1^, and Xueyun Gao^1,3,*^**

^1^CAS Key Laboratory for Biomedical Effects of Nanomaterials and Nanosafety, Institute of High Energy Physics, Chinese Academy of Sciences, Beijing 100049, China. ^2^University of Chinese Academy of Sciences, Beijing 100049, China. ^3^Department of Chemistry and Chemical Engineering, Beijing University of Technology, Beijing 100124, China. *^*^*Corresponding Authors: [gaoliang@ihep.ac.cn](mailto:gaoliang@ihep.ac.cn), [linazhao@ihep.ac.cn](mailto:linazhao@ihep.ac.cn) and [gaoxy@ihep.ac.cn](mailto:gaoxy@ihep.ac.cn).

**Table of Contents**

1. Binding analysis of Au_25_P2_9_ and Au_25_P3_9_ candidates to GPx-1

2. Hydrophobic interaction analysis of Au_25_P1_9_ binding to the active site of GPx-1

3. A549 cell viability assay after treated with peptides and peptide-Au clusters

Figure S1-S19

References

**1.** **Binding analysis of Au_25_P2_9_ and Au_25_P3_9_ candidates to GPx-1 surface**

The Au_25_P2_9_ candidate can bind to GPx-1 surface but deviate from the active site with 16.5 Å COM distance away. **In Figure S4a**, there are seven salt bridges constructed between four coating peptides (denoted as 1 to 4) of Au_25_P2_9_ and GPx-1 surface in the stable binding state at the 126^th^ ns, *i.e.* Asp406-Arg96 between peptide 1 and GPx-1 subunit A; Asp406-Lys84, Asp407-Lys110, Asp408-Lys110 between peptide 1 and GPx-1 subunit B; Asp406-Arg173 between peptide 2 and GPx-1 subunit A; Asp410-Arg10 between peptide 3 and GPx-1 subunit C; Asp407-Lys117 between peptide 4 and GPx-1 subunit C (positive charged residue in blue, and negative charged residue in red). The average numbers of salt bridges and hydrogen bonds are 9 and 12 between Au_25_P2_9_ and GPx-1 surface during the stable binding (50-130 ns), respectively. The correlation contact atoms averagely reach 126 (**Figure S4b**). The Au_25_P2_9_ is less stable binding to GPx-1 surface with less contact atoms than Au_25_P1_9_. On the other hand, the Au_25_P3_9_ candidate diffuses away from the active site region and binds to GPx-1 surface with 22.0 Å COM distance away from the aimed active site. **In Figure S4c**, there are six salt bridges formed between five coating peptides (denoted as 1 to 5) of Au_25_P3_9_ and GPx-1 surface in the stable binding configuration at the 126^th^ ns, *i.e.* Glu407-Arg178 between peptide 1 and GPx-1 subunit A; Glu407-Lys144 between peptide 2 and GPx-1 subunit A; C-terminal Gly412-Lys110 between peptide 2 and GPx-1 subunit B; Glu407-Lys84 between peptide 3 and GPx-1 subunit B; Glu410-Lys117 between peptide 4 and GPx-1 subunit B; C-terminal Gly412-Arg10 between peptide 5 and subunit C (positive charged residue in blue, and negative charged residue in red). The average numbers of salt bridges and hydrogen bonds are 9 and 9 between Au_25_P3_9_ and GPx-1 surface during the stable binding (50-130 ns), respectively. The correlation contact atoms averagely reach about 68 (**Figure S4d**). The Au_25_P3_9_ deviates from the active site with much looser binding to GPx-1 surface (much less contact atoms) than Au_25_P1_9_. The salt bridges and hydrogen bonds of Au_25_P2_9_ and Au_25_P3_9_ support them to bind to GPx-1 surface, but deviated from the active site.

**2. Hydrophobic interaction analysis of Au_25_P1_9_ binding to the active site of GPx-1**

In the stable configuration of Au_25_P1_9_ binding to the active site of GPx-1 (at the 126^th^ ns in MD simulation), There are three coating peptides (denoted as peptide 1 to 3) binding to GPx-1 surface by hydrophobic interactions as shown **in Figure S5**. Two interactions of them are Pro405-Val49, Val411-Leu180 between peptide 1 and GPx-1 subunit A. It is worthy to note, the hydrophobic sidechain of charged Glu in coating peptides can contribute much hydrophobic binding to the active site of GPx-1, *i.e.*, Glu406-Phe179 between peptide 2 and GPx-1 subunit A; Glu407 to 411 (denoted as Group1)-Ala138 of subunit A, Phe77, Pro120 to 123, Trp148, Pro150, Val156 of subunit C (denoted as Group2) between peptide 3 and GPx-1 surface. As a result, the longer hydrophobic sidechain of E (Glu) in P1 (than D, Asp in P2) support Au_25_P1_9_ more effective hydrophobic interactions to the uncharged residues distributed around the active site.

**3. A549 cell viability assay after treated with peptides and peptide-Au clusters**

We found free peptides cannot kill A549 cells **(Figure S19a)**, but peptide-Au clusters suppressed cell growth in a dose-dependent manner. At Au dose of 800 μM, cell viability was left 40%, 53% and 71% when it was treated with Au_25_P1_9_, Au_25_P2_9_, and Au_25_P3_9_, respectively **(Figure S19b)**. The results indicate free peptides have no effects on cell viability and Au_25_P1_9_ exhibits the best suppression capacity for A549 cell viability.


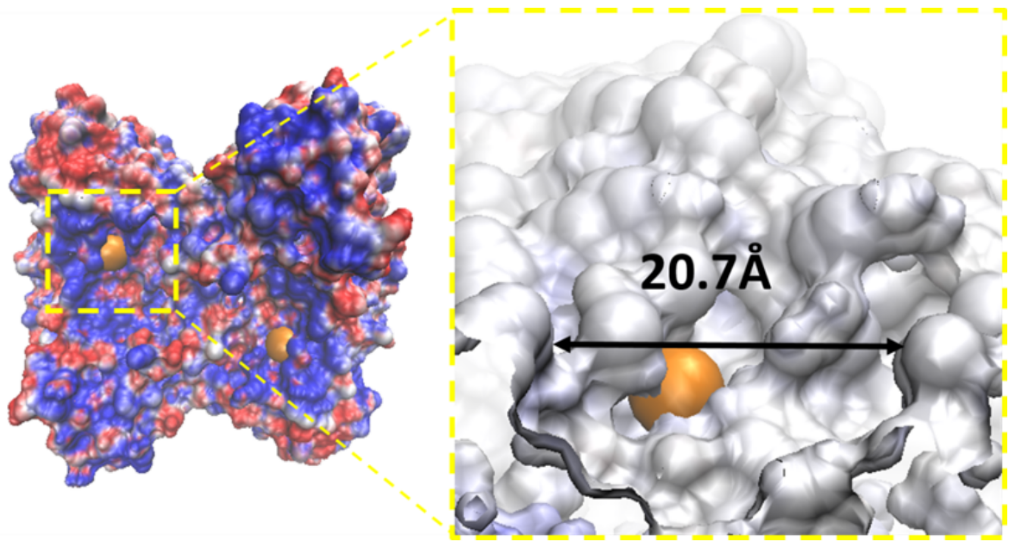


**Figure S1.** Surficial electrostatic potential distribution of GPx-1 (left) and concave structure of GPx-1 active site (right). Se atom in the active site Sec45 is highlighted in orange. Positive and negative charges are colored in blue and red. Uncharged distribution is in white.


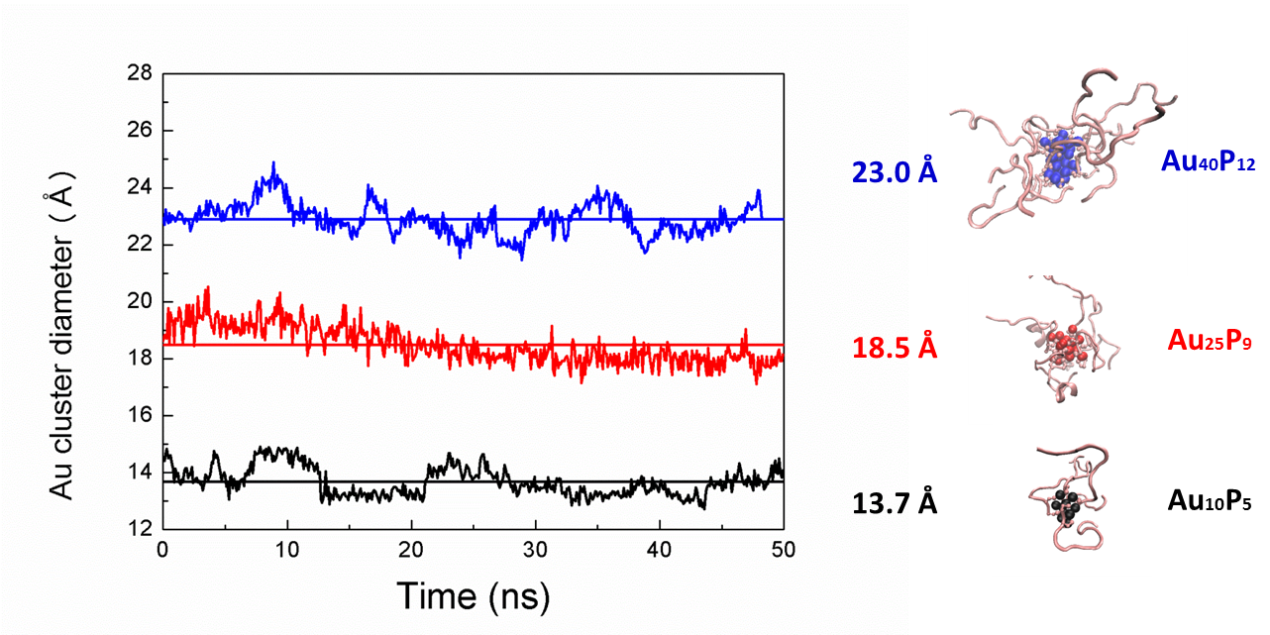


**Figure S2.** Diameters of typical peptide-Au clusters.


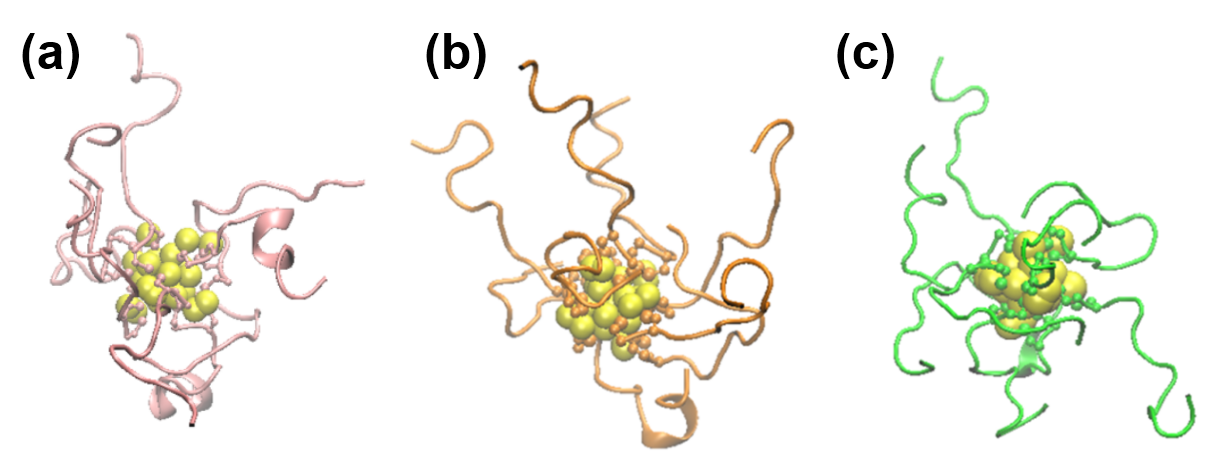


**Figure S3.** Representative configurations of (a) Au_25_P1_9_ (P1 in pink), (b) Au_25_P2_9_ (P2 in orange) and (c) Au_25_P3_9_ (P3 in green).


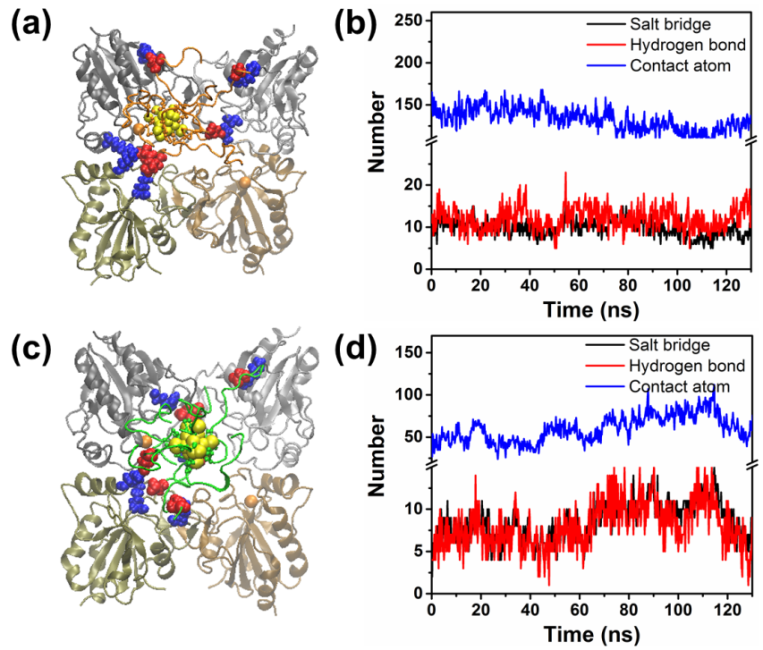


**Figure S4**. Binding analysis of Au_25_P2_9_ and Au_25_P3_9_ candidates to GPx-1 surface. (a) Salt bridge distribution between Au_25_P2_9_ (with orange peptides) candidate and GPx-1 surface in the stable configuration (at the 126^th^ ns in MD simulation). (b) The number of salt bridges (black), hydrogen bonds (red) and contact atoms (blue) between Au_25_P2_9_ candidate and GPx-1 surface. (c) Salt bridge distribution between Au_25_P3_9_ (with green peptides) candidate and GPx-1 surface in the stable configuration (at the 126^th^ ns in MD simulation). (d) The number of salt bridges (black), hydrogen bonds (red) and contact atoms (blue) between Au_25_P3_9_ candidate and GPx-1 surface. In (a) and (c), positive and negative charged residues building salt bridges are in blue and red respectively.


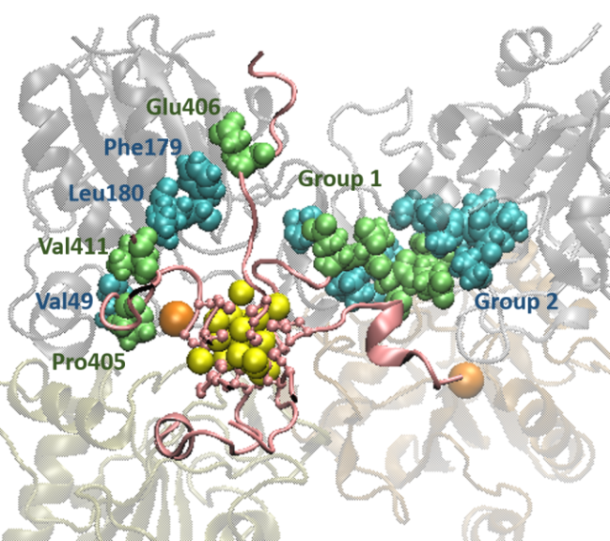


**Figure S5**. Hydrophobic interaction analysis of Au_25_P1_9_ binding to the active site of GPx-1 in the stable configuration (at the 126^th^ ns in MD simulation). The hydrophobic residues or hydrophobic parts of residues from Au_25_P1_9_ and GPx-1 are in green and blue, respectively. Se atom in Sec45 (active site) is in orange.


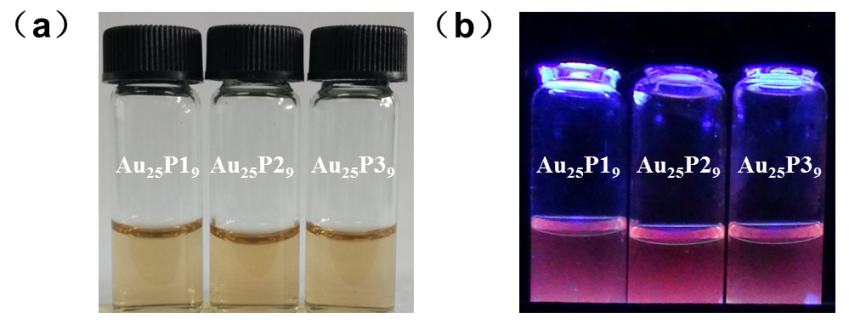


**Figure S6**. The picture of peptide-Au clusters solutions under visible-light (a) and UV irradiation at 365 nm (b).

**
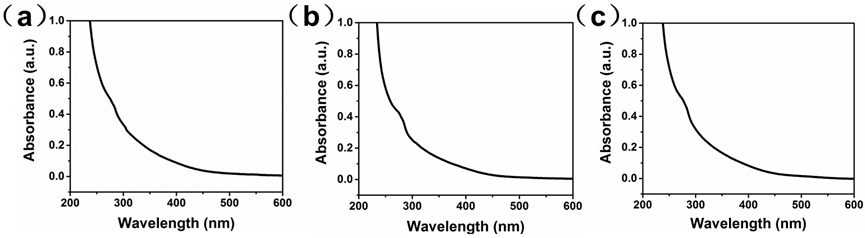
**

**Figure S7**. UV-Vis absorption spectra of Au_25_P1_9_ (a), Au_25_P2_9_ (b) and Au_25_P3_9_ (c).

**
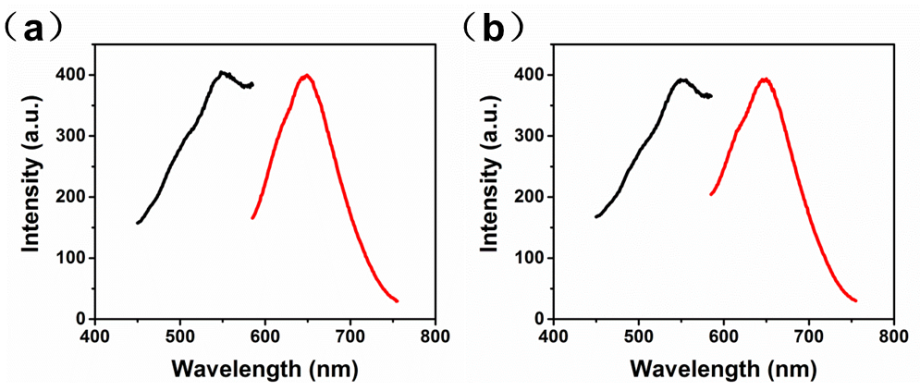
**

**Figure S8** Fluorescence excitation (black line) and emission (red line) spectra of Au_25_P2_9_ (a) and Au_25_P3_9_ (b).

**
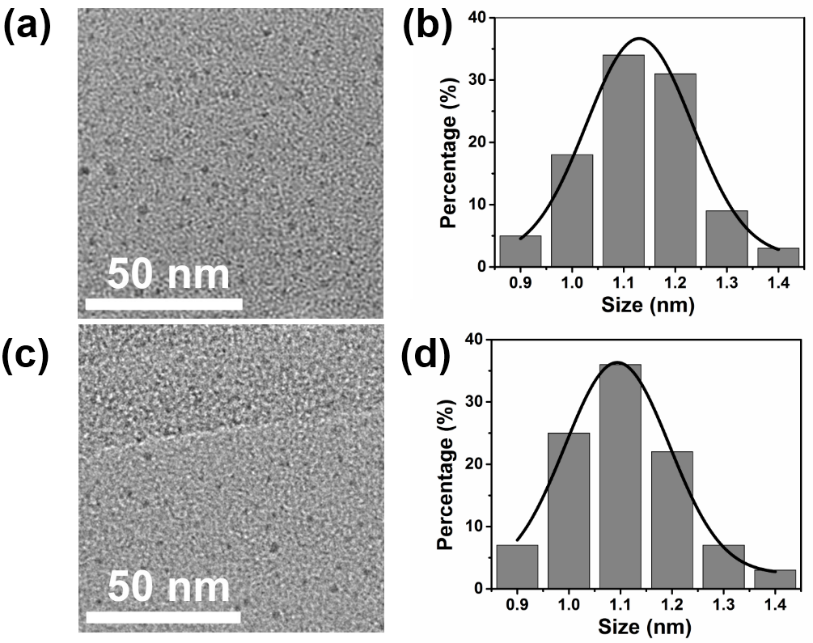
**

**Figure S9.** HRTEM images and diameter distribution histograms of Au_25_P2_9_ (a-b) and Au_25_P3_9_ (c-d). Diameter distribution histograms are from statistic results of 100 particles.


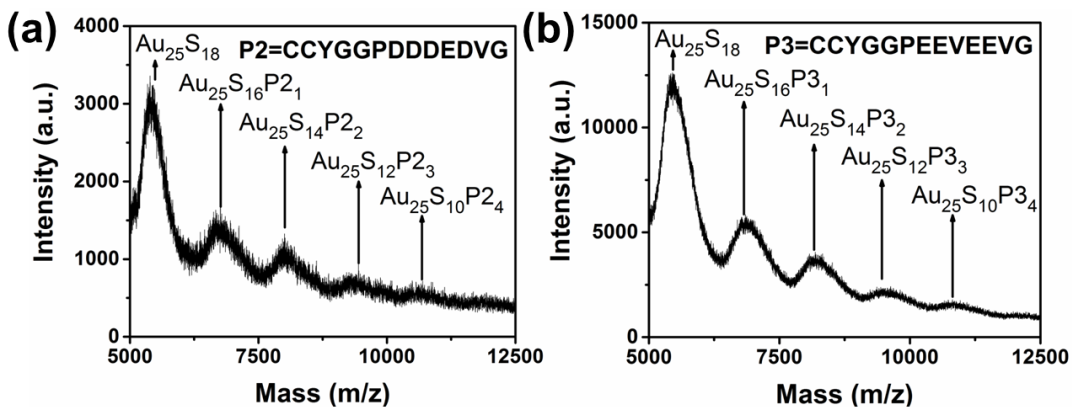


**Figure S10**. MALDI-TOF MS spectra of Au_25_P2_9_ (a) and Au_25_P3_9_ (b).


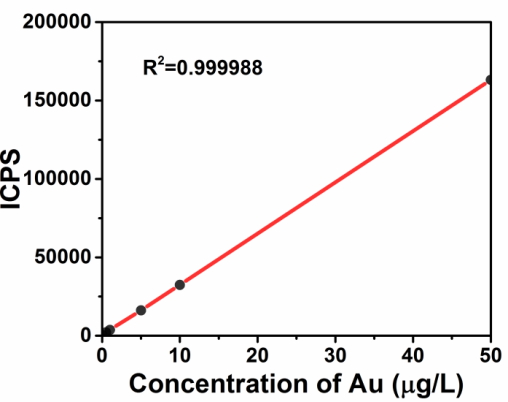


**Figure S11.** Calibration curve of Au standards by ICP-MS

**
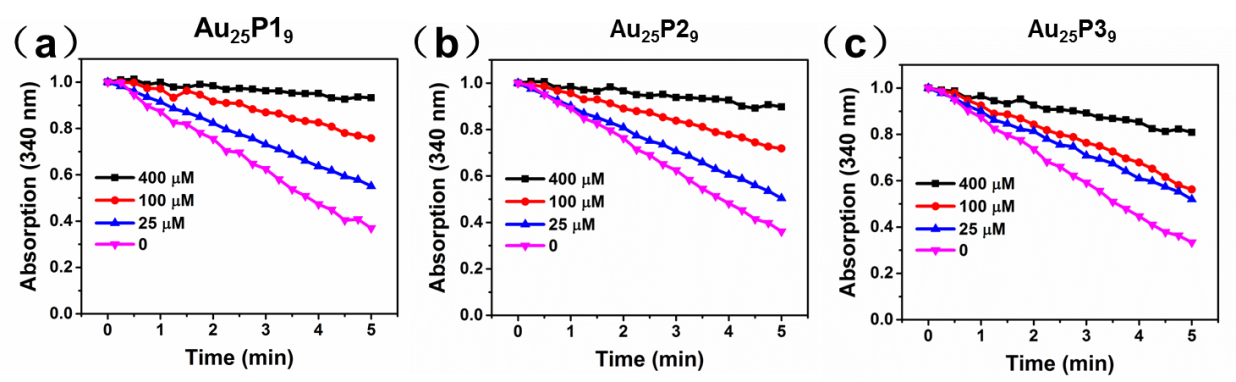
**

**Figure S12.** Peptide-Au clusters treated GPx-1 activity measured in buffer solution. The decrease in NADPH absorbance measured at 340 nm during the oxidation of NADPH to NADP is the indicative of GPx activity, which is in a dose dependent manner in tube experiments when treated with serial doses of peptide-Au clusters.


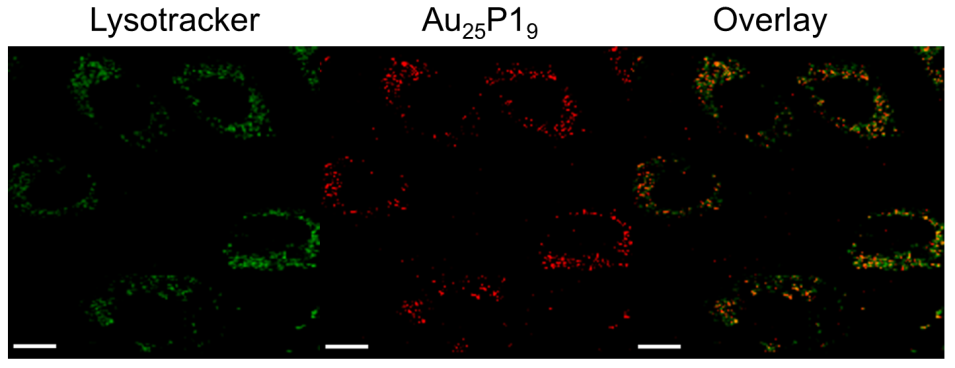


**Figure S13.** CLSM images of A549 cells treated with Au_25_P1_9_ and lysotracker green. Scale bar is ~11 µm.

**
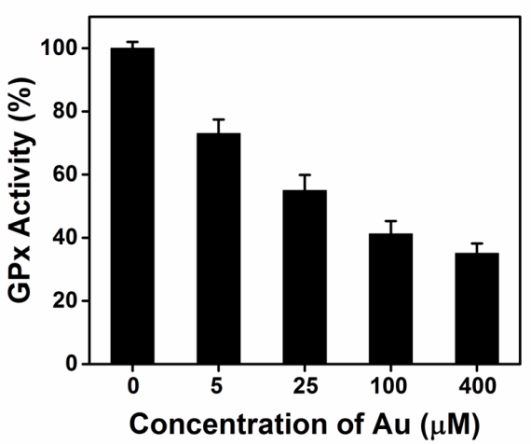
**

**Figure S14.** GPx-1 activity in A549 cell lysate suppressed by serial dose of Au_25_P1_9_. The activity of GPx-1 was also measured and the results showed that GPx-1 activity was suppressed via an Au dose dependent manner in cell lysate experiment.


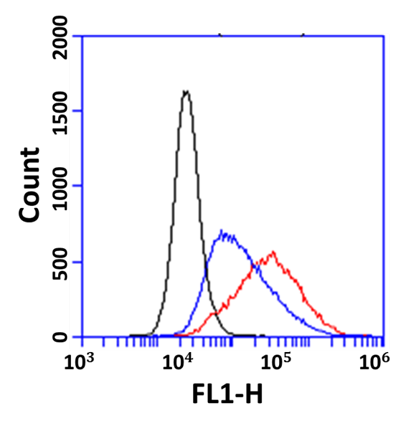


**Figure S15.** Flow cytometry analysis of ROS level of A549 cells treated and untreated with Au_25_P1_9_ (Au dose at 800 µM). Untreated cells, CM-H2DCFDA treated cells and Au_25_P1_9_ together with CM-H2DCFDA treated cells are in black (FL1-H, 2674), blue (FL1-H, 20088) and red (FL1-H, 43464), respectively, thus cellular ROS level increased to ~210% after treated with Au_25_P1_9_ (Au dose at 800 µM).


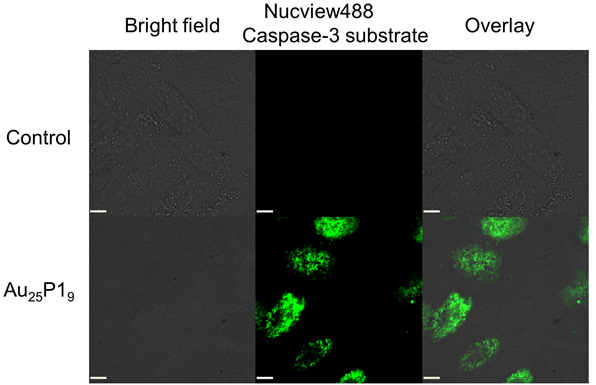


**Figure S16.** CLSM images of caspase-3 substrate in Au_25_P1_9_ treated A549 cells. Scale bar is ~11 µm

**
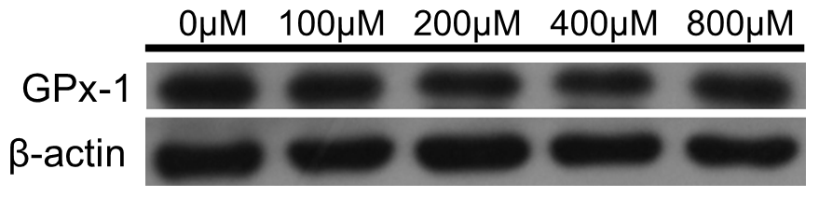
**

**Figure S17.** The expression level of GPx-1 extracted from Au_25_P1_9_ treated A549 cells under serial Au doses for 48 h.


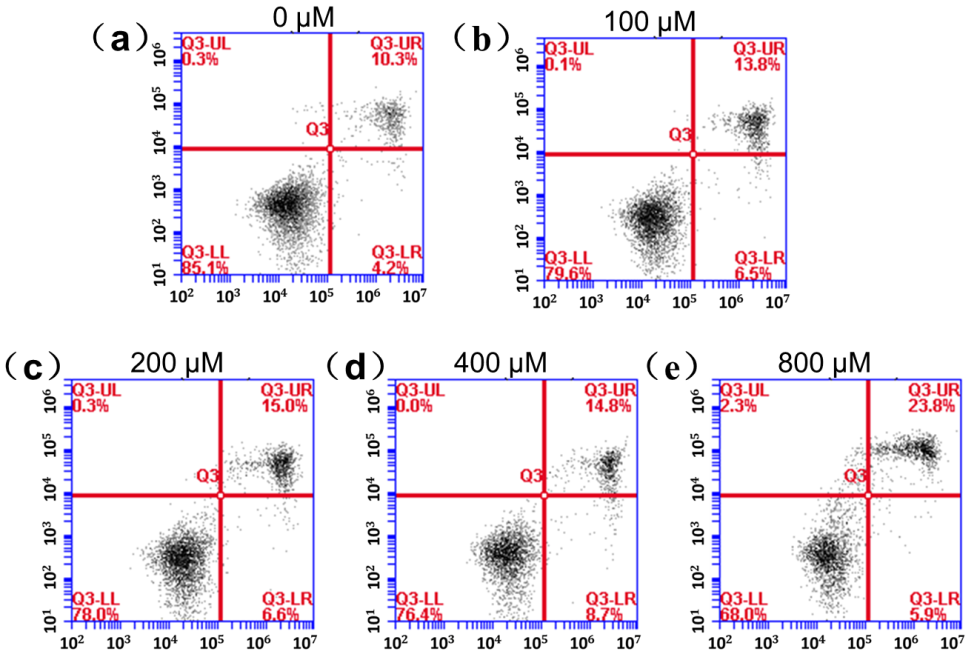


**Figure S18.** A549 cells apoptosis induced by Au_25_P1_9_, Au doses at 0μM (a), 100μM (b), 200μM (c), 400μM (d) and 800μM (e), respectively.


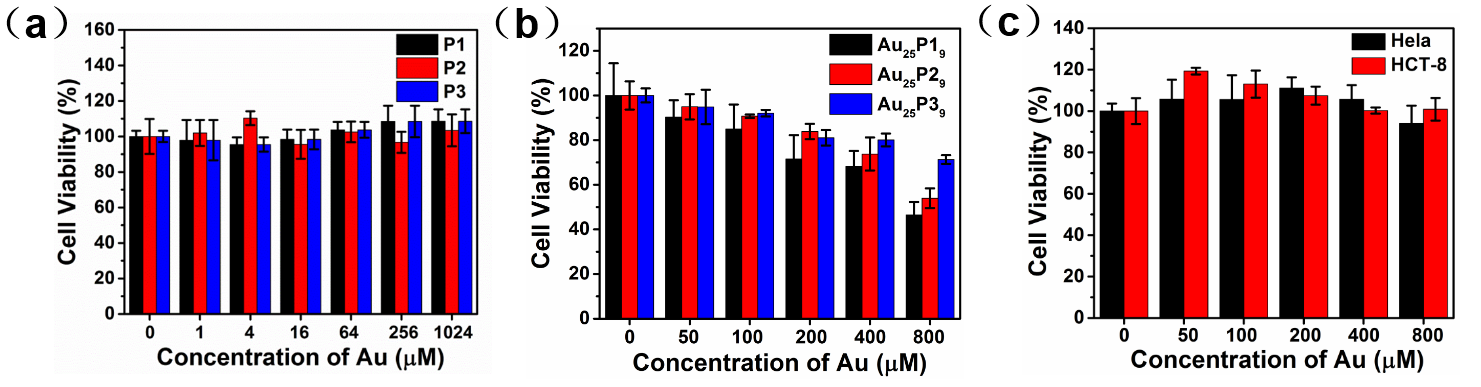


**Figure S19.** Cell viability of A549 cells after treated with peptides (a) and peptide-Au clusters (b). (c) Cell viability of Hela and HCT-8 cells after treated with Au_25_P1_9_.

**References**

1. Chaudiere, J. & Tappel, A.L. Interaction of gold(I) with the active site of selenium-glutathione peroxidase. *J. Inorg. Biochem.* **20**, 313-25 (1984).

2. Roberts, J.R. & Frank Shaw, C. Inhibition of Erythrocyte Selenium-Glutathione Peroxidase by Auranofin Analogues and Metabolites. *Biochem. Pharmacol.* **55**, 1291-99 (1998).
